# Geostatistical mapping of the seasonal spread of under-reported dengue cases in Bangladesh

**DOI:** 10.1371/journal.pntd.0006947

**Published:** 2018-11-15

**Authors:** Sifat Sharmin, Kathryn Glass, Elvina Viennet, David Harley

**Affiliations:** National Centre for Epidemiology and Population Health, Research School of Population Health, The Australian National University, Caberra, Australia; University of California, Davis, UNITED STATES

## Abstract

Geographical mapping of dengue in resource-limited settings is crucial for targeting control interventions but is challenging due to the problem of zero-inflation because many cases are not reported. We developed a negative binomial generalised linear mixed effect model accounting for zero-inflation, spatial, and temporal random effects to investigate the spatial variation in monthly dengue cases in Bangladesh. The model was fitted to the district-level (64 districts) monthly reported dengue cases aggregated over the period 2000 to 2009 and Bayesian inference was performed using the integrated nested Laplace approximation. We found that mean monthly temperature and its interaction with mean monthly diurnal temperature range, lagged by two months were significantly associated with dengue incidence. Mean monthly rainfall at two months lag was positively associated with dengue incidence. Densely populated districts and districts bordering India or Myanmar had higher incidence than others. The model estimated that 92% of the annual dengue cases occurred between August and September. Cases were identified across the country with 94% in the capital Dhaka (located almost in the middle of the country). Less than half of the affected districts reported cases as observed from the surveillance data. The proportion reported varied by month with a higher proportion reported in high-incidence districts, but dropped towards the end of high transmission season.

## Author summary

A better understanding of spatial and temporal variation in dengue risk is invaluable since it guides intervention strategies and facilitates effective health resource allocation. Transmission of dengue depends on the distribution and abundance of the mosquito vectors which are sensitive to climatic and environmental factors including temperature, rainfall, and population density. By modelling dengue-climate relationships, the burden of dengue can be estimated in locations where data on transmission are not available permitting identification of high-risk areas. This reveals the extent of under-reporting in dengue surveillance data in resource-limited countries which is essential to estimating the changing burden of dengue.

## Introduction

**Fig 1:**
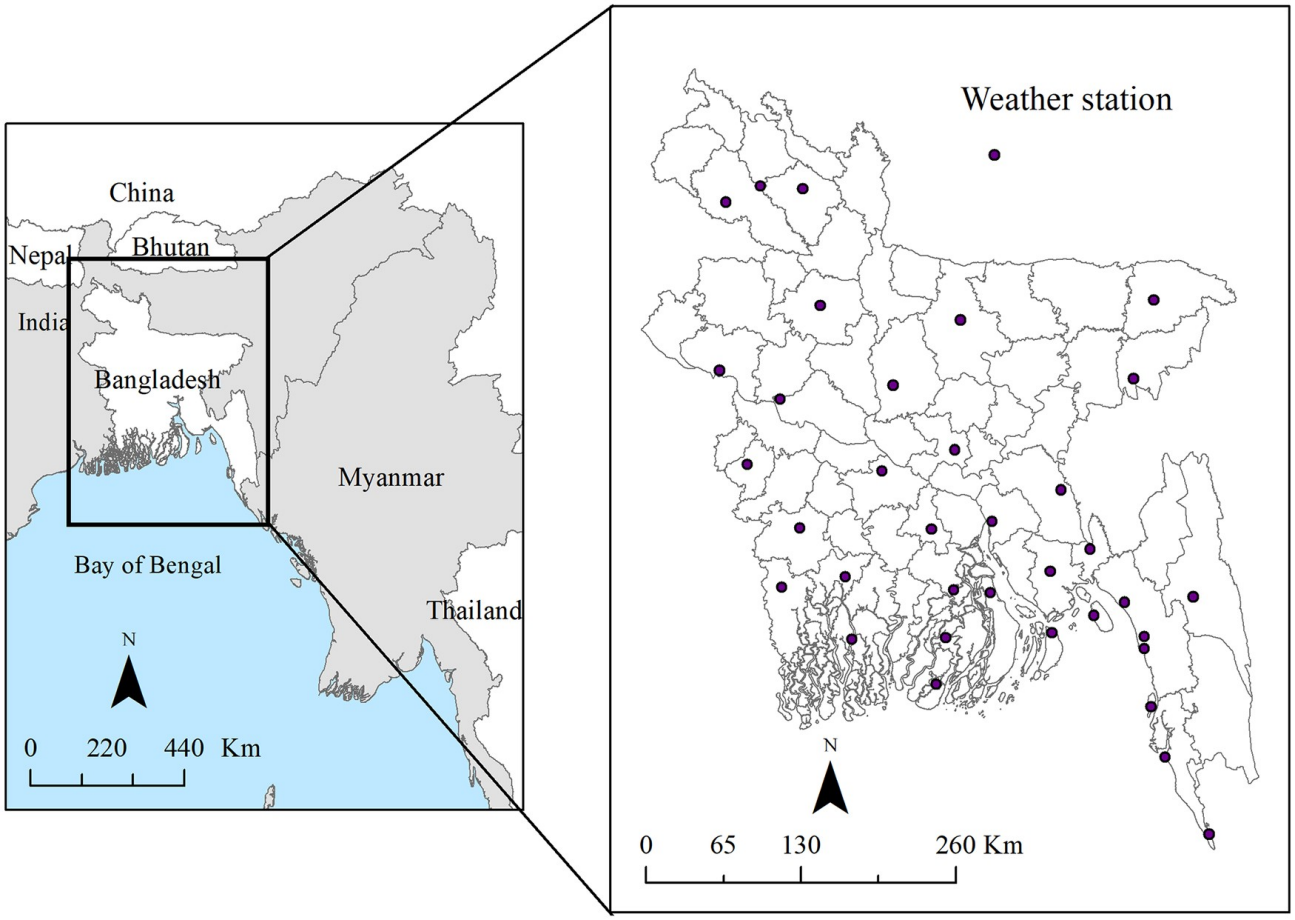
Location of Bangladesh (left) and district-level map of Bangladesh with 35 weather stations marked with a black dot (right). The figure was produced using ArcGIS 10.5.1 (ESRI, Redlands, California).

Dengue is a neglected tropical disease caused by the dengue virus (DENV) and is transmitted by female *Aedes* mosquitoes, predominantly *Aedes aegypti* and *Aedes albopictus*. The severe forms of the disease are potentially fatal. The World Health Organization (WHO) estimates that about 52% of the people at risk of dengue worldwide live in 10 countries of the WHO South-East Asia Region [1]. Bangladesh, located in South Asia and surrounded by India and Myanmar (Fig 1) where dengue is endemic, experienced its first epidemic of dengue in 2000 [2]. Since then cases have been reported every year, most commonly among adults and older children living in metropolitan cities [3]. Of the 64 Bangladeshi districts (Bangladesh’s second largest administrative unit), 29 reported dengue cases between 2000 and 2009, with Dhaka consistently reporting the largest number of cases [4]. Heterogeneity in the distribution of hospitals across the country and differentials in treatment-seeking behaviour based on location are likely to cause under-reporting [3]. However, despite no effective control program being introduced and no changes in surveillance until 2010 when serological confirmation was mandated for case reporting, nationally reported cases have declined since 2002 [3]. This might be partially attributable to increased prevalence of immunity and reduction in mosquito breeding sites resulting from public awareness [3]. However, there is considerable under-reporting inherent in the passive hospital-based surveillance system [3]. A study investigating the global distribution of dengue burden estimated an average of 4,097,833 symptomatic infections (95% Bayesian credible interval: 2,952,879–5,608,456) occurred in Bangladesh in 2010 [5] but only 409 were reported to authorities [4].

Climatic factors (mainly temperature and rainfall) influence the survival and development rate of vector and virus. The aquatic larval and pupal stages of the *Aedes* mosquitoes require fresh water. Outdoor artificial containers filled with rain water serve as breeding sites [6]. Average temperature, as well as diurnal temperature range (DTR, the difference between daily maximum and minimum temperature), influence mosquito development, the mosquito biting rate, the extrinsic incubation period, and vector-virus-host interaction [7–10].

Climatic similarity and the movement of viraemic individuals in geographically neighbouring areas introduce spatial correlation in dengue incidence [11,12] and might lead to spurious model-based incidence estimates if ignored. Bayesian geostatistical modelling approaches are powerful for disease mapping, explicitly accounting for spatial correlation in disease data while incorporating uncertainty in data and model parameters [13,14]. Within a Bayesian paradigm, inference about model parameters is based on the posterior distribution derived from the combination of data and pre-existing knowledge of parameter values, and therefore does not require a large sample size assumption as does the frequentist approach. Therefore, more robust estimates can be obtained when the disease is rare [15] or data on case numbers are limited.

Regional variation in dengue must be studied to allocate resources proportionate to burden but this has not been done in Bangladesh. The only spatial mapping study of dengue in Bangladesh identified Dhaka as the most likely cluster for dengue transmission during 2000–2009 with a small number of secondary clusters in the southern part of the country in 2000 [16]. However, under-reporting was not accounted for, nor were potential causes of geographical variation in dengue transmission (such as climate and socio-demographic factors) considered. Our aim was to produce maps of the monthly spatial variation in dengue incidence at the district-level in Bangladesh in relation to climatic and demographic factors with adjustment for under-reporting. The resulting maps can facilitate the efficient distribution of vector control interventions to areas of highest need at the appropriate time. The method is useful for identifying locations where DENV transmission occurs but incidence data are lacking.

## Materials and methods

### Ethics statement

The study was approved by The Australian National University Human Research Ethics Committee. National dengue surveillance data were anonymised.

### Study area

Bangladesh has a hot, humid, tropical climate with monsoons occurring during June to September. Monsoon rainfall, about four-fifths of the mean annual rainfall, ranges from 1,527mm in the west to 4,197mm in the east, and the mean monthly monsoon temperature of 29°C averaged across the country is generally suitable for dengue transmission.

High population density (964 people per square kilometre in 2011) and unplanned urbanisation leading to over-crowding in divisional cities with inadequate water supply, and inefficient drainage and waste disposal increase risk for dengue since the vector mosquitoes breed in water storage containers in and around houses [6,17,18].

### Data

Dengue notification data, consisting of suspected, probable, and confirmed cases reported to the Directorate General of Health Services between January 2000 and December 2009 were analysed. Under-ascertainment of symptomatic dengue cases is highly likely since the passive surveillance system only reports cases admitted to hospital [3]. Daily rainfall (mm), and minimum and maximum temperatures (°C) measured from 35 stations were sourced from the Bangladesh Meteorological Department with around 2, 3, and 3% missing data, respectively. Diurnal temperature range (DTR) was calculated from the daily maximum and minimum temperatures. Weather values for days with missing data were filled by averaging data from adjacent days. Monthly averages were then calculated from daily records. Missing values for months were supplemented with the average of non-missing values from three neighbouring stations. Bayesian kriging [19] was used to interpolate weather values in districts without a station. Mean monthly rainfall, mean monthly temperature, mean monthly DTR, and monthly total dengue cases over the 10 years were then aggregated by month and used in the model development discussed below.

The population density (people/km^2^) for each district was estimated by dividing the district population by the district area (km^2^). The monthly population of each district were calculated by linear interpolation between population estimates from the 1991, 2001, and 2011 census data of the Bangladesh Bureau of Statistics [20].

### Model formulation

More than half of Bangladeshi districts did not report any dengue cases during the study period 2000 to 2009. It was unclear whether these missing values corresponded to true zeros or a lack of reporting, so we used a zero-inflated model which allows for two different interpretations for the occurrence of zero cases; either no case occurred, or cases occurred but were not reported.

To model the spatio-temporal pattern of dengue, district-wise dengue notification data aggregated by month over the period 2000 to 2009 were modelled via a negative binomial generalised linear mixed effect model with a logarithmic link function and the population of the districts as an offset. Let *y*_*it*_ be the number of reported dengue cases for the *i*^*th*^ (*i* = 1, 2,…, 64) district in the *t*^*th*^ (*t* = 1, 2,…, 12) month. Preliminary non-spatial analysis indicated that the following factors should be included in the analysis: mean monthly temperature, mean monthly DTR, interaction between mean monthly temperature and DTR, and mean monthly rainfall at lag one and two months, and population density [21]. The variable “Border” indicating whether a district bordered India or Myanmar was included in the model. This variable was used as a proxy for movement across borders with neighbouring dengue endemic countries. An indicator for outbreak months with case numbers exceeding the 10-year mean plus two standard deviations was added. Population age structure which is approximately the same across the districts was not considered [22]. The generalised linear mixed effect model is specified by:

$$\begin{array}{ll} \text{log}\left(\mu_{it}\right) & =\text{log}\left(Population_{it}\right)+\alpha +\beta_{1}T_{i,t-1}+\beta_{2}T_{i,t-2}+\beta_{3}DTR_{i,t-1}+\beta_{4}DTR_{i,t-2}\\ & +\beta_{5}(T_{i,t-1}*DTR_{i,t-1})+\beta_{6}(T_{i,t-2}*DTR_{i,t-2})+\beta_{7}R_{i,t-1}+\beta_{8}R_{i,t-2}\\ & +\beta_{9}*Border_{i}+\beta_{10}*Outbreak_{it}+\beta_{11}*Popden_{it}+\emptyset_{i}+AR{\left(1\right)}_{t};\\ & i=1,2,\ldots ,64\;and\;t=1,2,\ldots ,12 \end{array}$$

where *μ*_*it*_ denotes the mean dengue counts for the *i*^*th*^ district in the *t*^*th*^ month. *T*, *DTR*, (*T* * *DTR*), *and R*, are the district wise mean monthly values of temperature, diurnal temperature range, interaction between temperature and diurnal temperature range, and rainfall. *AR*(1)_*t*_ is a first order autoregressive month effect that captures the correlation in dengue cases between consecutive months. ∅_*i*_ represents the spatially structured random effects at district-level that take into account the spatial dependency in dengue cases by assuming a Matérn covariance using the stochastic partial differential equations (SPDE) approach [23]. All continuous covariates were standardised (zero mean and variance one) to ensure that the influence of each covariate parameter was comparable. The model fitting was done using the R-package Integrated Nested Laplace Approximation (INLA) [24].

Following a Bayesian model specification, prior distributions were assigned to model parameters. Independent diffuse Gaussian priors (with mean 0, precision 1 × 10^−3^) were chosen for the intercept (*α*) and regression coefficients (β**)** to allow the data to predominate in calculating the posterior distributions. The precision parameter for the random effect was assigned a logGamma (1, 0.00005) prior.

The deviance information criteria (DIC) was used to check the goodness of fit of three zero-inflated negative binomial models (ZINB) developed sequentially as a temporal model, a spatio-temporal random effects model, and a spatio-temporal model without any fixed effect covariates. A low DIC value is indicative of the best trade-off between model fit and complexity of the model.

## Results

**Fig 2:**
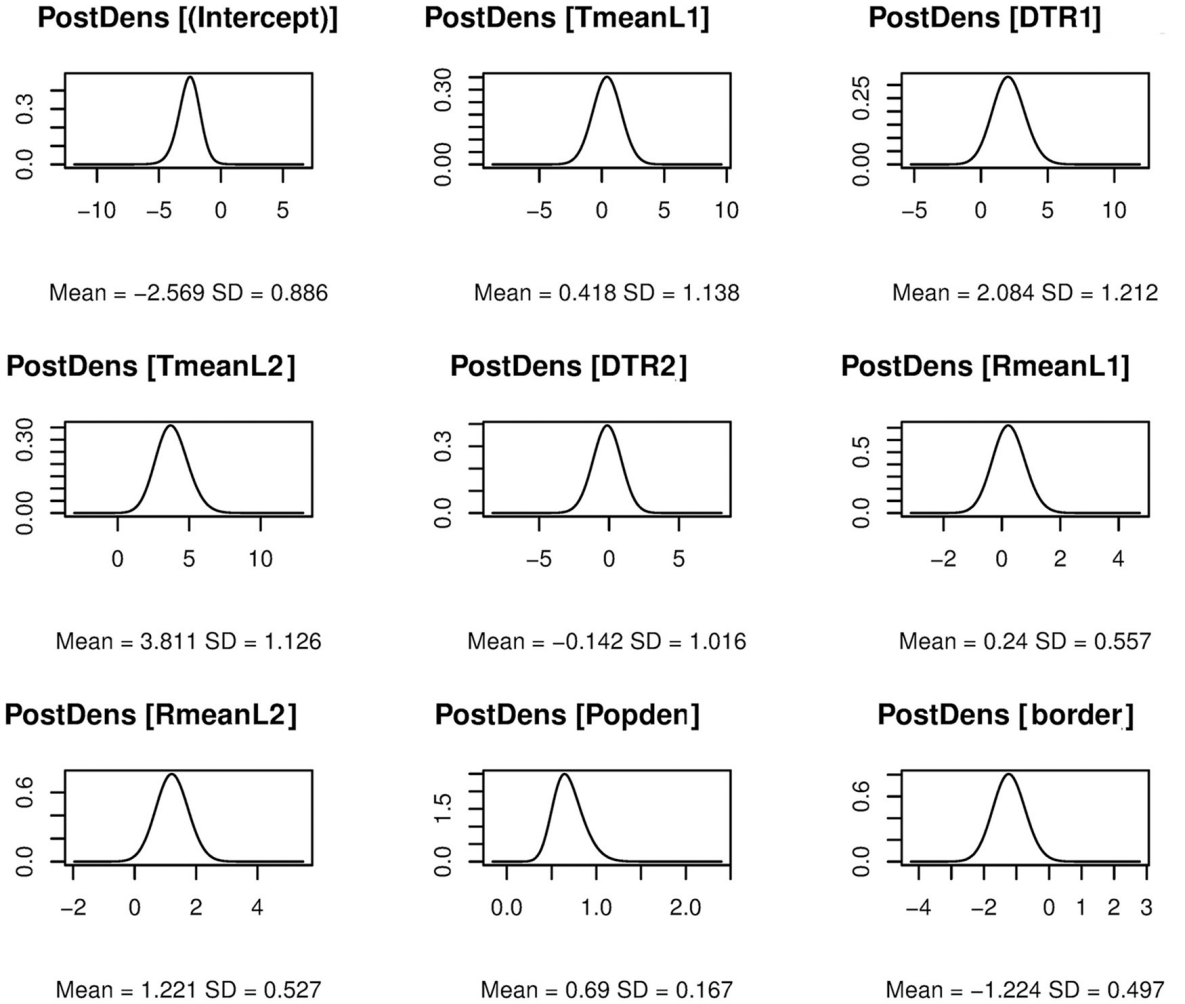
Posterior density plots of fixed effect parameters derived from the spatio-temporal full model. The figure was produced using R-package INLA.

Table 1 displays parameter estimates from the three ZINB models. The spatio-temporal full model fitted the data best as evidenced by the lowest DIC (1079.44 vs 1099.54 and 1132.55). Of all the climatic variables, mean monthly temperature, its interaction with DTR, and mean monthly rainfall at lag two months showed significant associations with dengue incidence. Average temperature had a positive relationship with dengue (*β*_2_ = 3.81; 95% highest posterior density [HPD] credible interval [CrI]: 1.63, 6.04), while it’s negative interaction (*β*_6_ = -1.73; 95% HPD CrI: -3.13, -0.36) with DTR indicates that increasing temperature and decreasing DTR is associated with increased dengue incidence. Mean monthly rainfall at two months lag showed a positive relationship (*β*_8_ = 1.22; 95% HPD CrI: 0.19, 2.26) with dengue. Population density was significantly (*β*_11_ = 0.69; 95% HPD CrI: 0.38, 1.03) associated with dengue. Districts not adjoining the borders with India or Myanmar were found to have significantly lower incidence compared with adjacent districts. The posterior density plots of fixed effect covariates are shown in Fig 2.

**Table 1:** Posterior mean and corresponding 95% highest posterior density (HPD) credible intervals (CrI) for parameters of the three zero-inflated negative binomial models.

Parameters	Temporal model, Posterior mean (95% HPD CrI)	Spatio-temporal full model, Posterior mean (95% HPD CrI)	Spatio-temporal model without fixed effect covariates, Posterior mean (95% HPD CrI)
Temperature lagged 1 month	-1.52 (-3.69, 0.60)	0.42 (-1.82, 2.65)	
DTR lagged 1 month	1.01 (-1.18, 3.25)	2.08 (-0.28, 4.47)	
(Temperature*DTR) lagged 1 month	0.04 (-1.25, 1.34)	-0.88 (-2.26, 0.50)	
Rainfall lagged 1 month	0.03 (-1.05, 1.12)	0.24 (-0.85, 1.34)	
Temperature lagged 2 months	6.79 (4.25, 9.47)	3.81 (1.63, 6.04)	
DTR lagged 2 months	1.57 (-0.16, 3.32)	-0.14 (-2.14, 1.84)	
(Temperature*DTR) lagged 2 months	-3.34 (-4.87, -1.86)	-1.73 (-3.13, -0.36)	
Rainfall lagged 2 months	1.17 (0.18, 2.17)	1.22 (0.19, 2.26)	
Population density	0.68 (0.24, 1.18)	0.69 (0.38, 1.03)	
Outbreak month			
Yes	2.80 (0.95, 4.85)	3.20 (1.37, 5.17)	
No	Ref	Ref	
Border			
Yes	Ref	Ref	
No	-1.86 (-3.03, -0.69)	-1.22 (-2.20, -0.25)	
Zero-inflation parameter	0.06 (0.05, 0.09)	0.08 (0.06, 0.10)	0.06 (0.04, 0.08)
Kappa		0.28 (0.20, 0.39)	0.31 (0.11, 0.80)
Tau		1.50 (1.03, 3.18)	12.19 (2.44, 272.06)
Moran’s Index:		0.12 (p-value<0.0001)	0.14 (p-value<0.0001)
DIC	1099.54	1079.44	1132.55

* interaction between two variables

A zero-inflation parameter with mean 0.08 (95% HPD CrI: 0.06, 0.10) confirms significant zero-inflation in reported dengue data. The Moran’s index (0.12; *p*-value<0.0001) provided significant evidence against the null hypothesis of zero spatial autocorrelation in dengue cases in Bangladesh. Significance of the spatial effects were evident from the estimates of tau and kappa.

**Fig 3:**
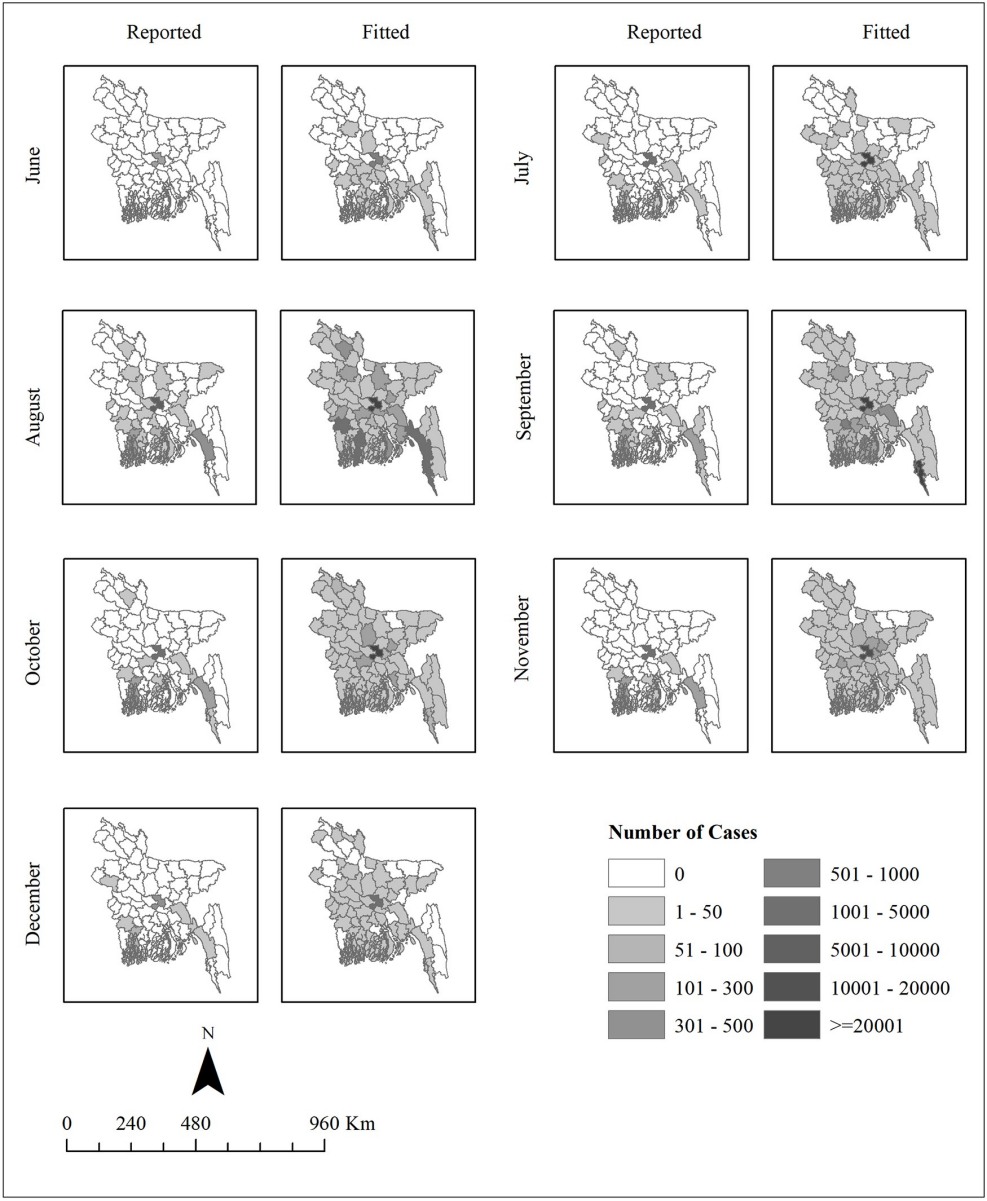
Spatial pattern in monthly aggregated dengue cases reported between 2000 and 2009 and average number of fitted dengue cases, estimated by the spatio-temporal full model. **Only 0.01% of cases were estimated outside the period June-December and are not shown here**. The figure was produced using ArcGIS 10.5.1 (ESRI, Redlands, California).

Fig 3 shows the spatial variation in monthly aggregated dengue cases reported over 2000–2009, compared with the corresponding fitted values obtained from the spatio-temporal full model. The model clearly captures the spatial variation between months with an increasing trend in case numbers from June with the highest in August. Southern Bangladesh observes higher transmission than the northern part of the country. The model identified several districts (mostly in the northern part of the country) with modelled transmission but without any reported cases.

**Fig 4:**
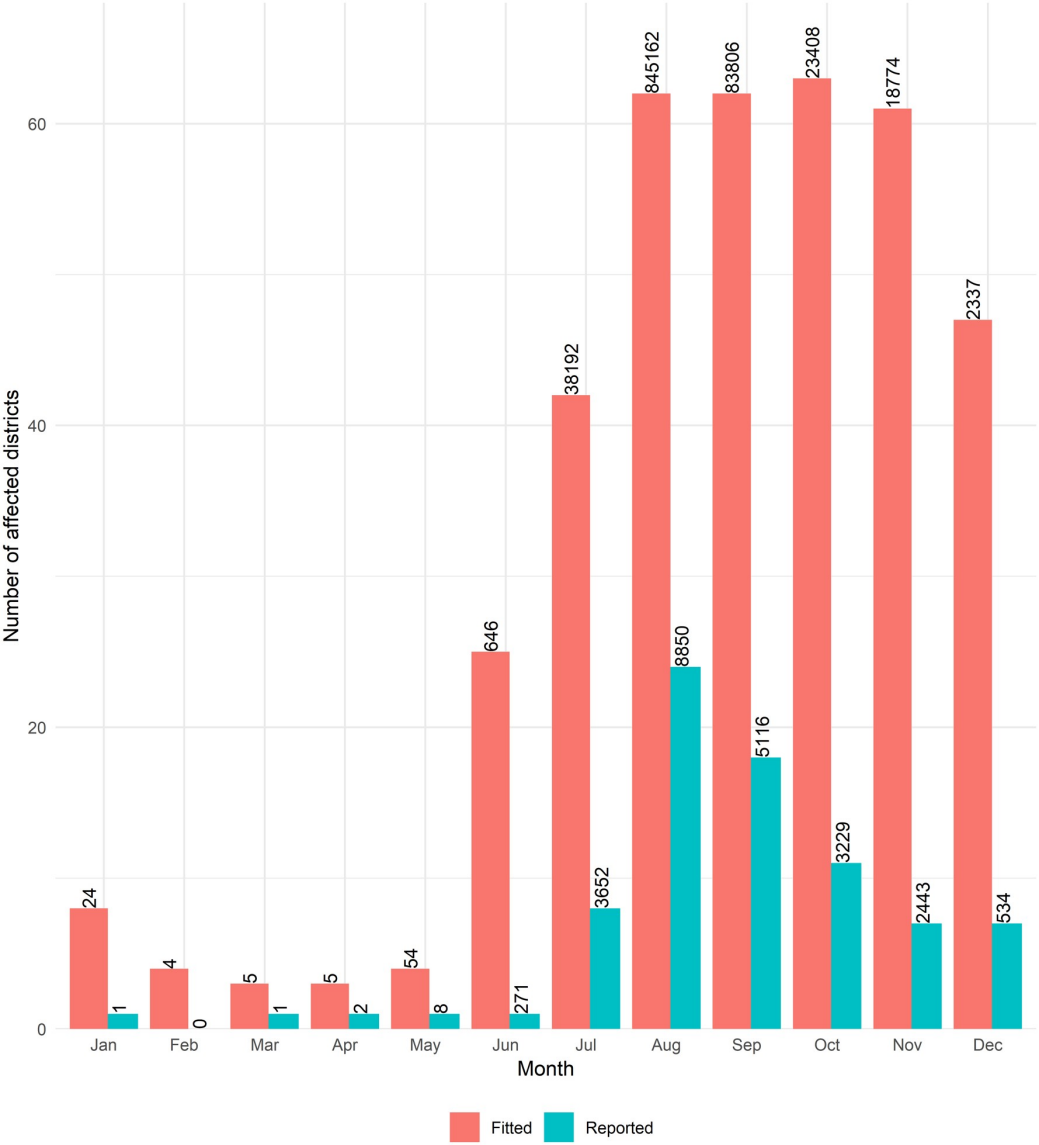
Paired bar chart to show the number of districts that reported dengue cases during 2000–2009 and the number of districts with transmission estimated from the spatio-temporal full model. **The numbers on top of the bars represent total monthly number of cases reported and estimated by the model**. The figure was produced using R-package ggplot2.

Dengue transmission fluctuated over the months with 92% of estimated annual cases occurring in August and September. During this period, almost all the districts across the country were estimated to have cases, although 94% of national total were in Dhaka. National surveillance data reported cases from less than half of the affected districts estimated by the model (Fig 4).

**Fig 5:**
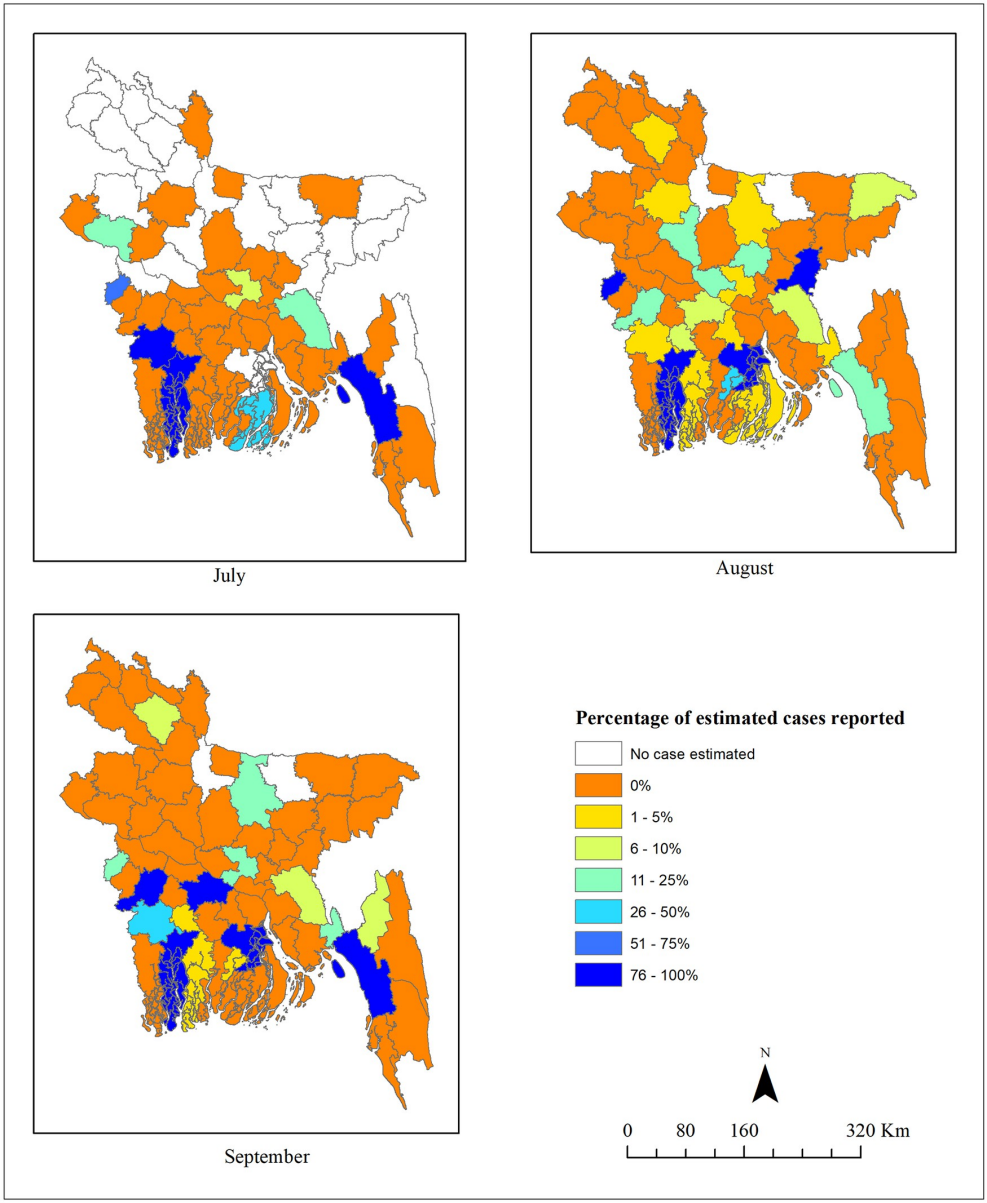
Monthly spatial variation in the percentage of estimated total cases that were reported to the surveillance system, calculated as (reported case numbers/mean estimate of total case numbers estimated by the model)*100. The figure was produced using ArcGIS 10.5.1 (ESRI, Redlands, California).

Fig 5 shows the spatial variation in the percentage of estimated cases reported by month (July-September). In general, districts with higher case numbers had a higher percentage of estimated cases reported compared to the districts with fewer case numbers during the high transmission months of August and September.

## Discussion

We present the first model-based estimates of the monthly geographical distribution of dengue cases in Bangladesh. In national surveillance data more than half the districts reported zero cases but we believe disease occurred but was not reported. To address this zero-inflation, a ZINB model was developed. The spatio-temporal full model identified that increasing mean monthly temperature and decreasing DTR at lag two months were significantly associated with high incidence of dengue. The inverse relationship between temperature and DTR in our study corroborates previous observations [10,25]. Our analysis also suggested a positive association between dengue and mean monthly rainfall at two months lag. Several studies reported that increasing mean monthly temperature and rainfall significantly increase dengue risk at different time lags [26–28], reflecting the delays in the impact of weather on mosquito populations and subsequent changes in transmission patterns. Population density was found to be significantly associated with dengue risk. High transmission of dengue in densely populated areas has been reported in the literature [29,30]. Incidence was higher in districts bordering India or Myanmar, perhaps due to cross-border migration from endemic countries to Bangladesh [31].

The model identified highest transmission during August and September (92% of estimated annual cases). Transmission is spatially heterogeneous, with a higher number of cases estimated in the south compared to the northern districts, and the highest in Dhaka. An earlier study investigated space-time clusters of dengue transmission in Bangladesh from 2000 to 2009 and reported Dhaka as the most likely cluster throughout the study period, with a small number of secondary clusters in the south of the country in 2000 [16]. June-November was reported as the high transmission season when all the clusters were identified [16].

Our model also identified districts with transmission that went unreported; during the high transmission month of August, cases were reported from less than 50% of the affected districts. We estimated that the reporting percentage (the percentage of all cases reported to the surveillance system) also varies by month. During the high transmission months, reporting is generally higher in districts with high compared to those with low transmission. However, towards the end of the high transmission season, proportion reported declined in majority of districts presumably due to the lower case numbers.

A study measuring global burden of dengue estimated 4,097,833 symptomatic infections (95% Bayesian credible interval: 2,952,879–5,608,456) in Bangladesh in 2010 [5] which differs considerably from our annual estimate presumably due to the differences in data and modelling strategy. A two-stage analytical approach was taken by Bhatt et al [5] where a boosted regression tree approach was adopted to estimate the relationship between the probability of occurrence of a dengue infection and the environmental conditions sampled at each study site. Covariates included vegetation index, indicators of urbanisation and relative poverty, and an urban accessibility metric in addition to temperature and precipitation [5]. Annual estimates of infections were obtained from a hierarchical Bayesian model estimating the relationship between longitudinal incidence data from 54 cohort studies and the previously generated probability of occurrence of dengue infection [5]. The estimated apparent dengue infections referred to any infection encompassing any disruption to the daily routine of the infected individual [5], whereas our estimates are calculated based on hospital admitted patients and therefore not representative of all symptomatic infections. Sparse data points and lack of cohort studies across a range of transmission intensities in the Indian subcontinent could be responsible for the large uncertainties associated with the Bhatt et al’s estimates in this region [5].

The strength of our model is the ability to generate estimates of dengue in areas with suspected under-detection. Relatively low case numbers indicate the potential of active transmission of the disease in a district which is crucial in the absence of information regarding the mosquito vector population. This is also indicative of increased transmission risk across the neighbouring districts resulting from inter-district movements of viraemic individuals. The assumption that transmission is governed by climatic suitability is reasonable, especially in the absence of effective public health strategies including mosquito control program. Our model was incapable of capturing the inter-annual variation in dengue and climate variables due to the aggregation of monthly counts over the study period which was required to model the monthly spread of dengue across the country.

In conclusion, our study provides model-based estimates of spatial variation in monthly dengue cases across Bangladesh without compromising data for model fit. We believe that our findings provide a valuable assessment of the national dengue situation accounting for under-reporting. This study will contribute important information for prioritising and targeting dengue control and elimination interventions across Bangladesh.
